# Nursing after the Operation for Hare Lip

**Published:** 1859-04

**Authors:** 


### ARTICLE XIX.

### *Nursing after the Operation for Hare Lip.*

At a recent meeting of the Boston Society for Medical Improvement, Dr. Warren said that some years since he had advocated, in this society, the propriety of allowing the child to nurse after the operation for hare-lip, and had shown, by a number of cases, that this could be done without endangering union of the parts. Since that time he had followed this practice, and allowed all the patients, who were able to nurse, to do so. The advantages were, the greater quiet of the patient, and the less liability to disturbance of the bowels which almost always follows the change of diet, and which once or twice he had known to proceed to such an extent, as to defeat the operation. Dr. W. further remarked that he still preferred the suture to the needle, the latter, in spite of the greatest precaution, almost always leaving an ulceration, and being more troublesome to withdraw. On examining the interior of a hare-lip, after the skin has been approximated by sutures, Dr. W. said that a gaping wound will almost always be found, and one edge, generally the outer, projecting much beyond the other. The consequence is, a long delay in union, after everything externally appears sound. To obviate this, he had been in the habit, after making the lower suture, and before cutting off the ends of the threads, to evert the lip with them, and make a suture on the inside. These threads being now cut off short, the suture takes care of itself. For dressing, he had generally used a bit of wet linen, of a single thickness, the edges being unravelled, so as to make it, when moistened, the more adherent to the face—this should be frequently removed by the mother when it becomes dry, and restored. It almost entirely prevents the suppuration about the stiches.

[*Boston Med. and Surg. Jour.*

